# Effects of masks on the transmission of infectious diseases

**DOI:** 10.1186/s13662-021-03321-z

**Published:** 2021-03-18

**Authors:** Lili Han, Qiuhui Pan, Baolin Kang, Mingfeng He

**Affiliations:** 1 School of Mathematical Sciences, Dalian University of Technology, Dalian, China; 2 School of Innovation and Entrepreneurship, Dalian University of Technology, Dalian, China; 3 College of Mathematics and Information Science, Anshan Normal University, Anshan, China

**Keywords:** Differential equation model, Basic reproduction number, Asymptomatic virus carriers, Duration of the epidemic, Numerical results

## Abstract

In the present paper, based on the conditions that asymptomatic virus carriers are contagious and all symptomatic infected individuals wear masks, we study the impact of wearing masks in the susceptible and the asymptomatic virus carriers on the spread of infectious diseases by developing a differential equation model. At first, we give the existence, uniqueness, boundedness, and positivity of the solution as well as the basic reproduction number $R_{0}$ for the established model. Then, for two cases of wearing masks in the susceptible and the asymptomatic virus carriers where the proportion of wearing masks is fixed and the proportion of wearing masks changes with time, the results of the numerical simulation are shown in a series of pictures, and quantitative description of effects of the proportion of the population wearing masks, the protective effect of masks, and the time when they start wearing masks on the epidemic is given. Our results show that under the situation that the proportion of wearing masks is positively related to the confirmed new cases and new deaths, though the proportion will be close to 1 during the high incidence of patients, the effect on controlling the spread of such infectious diseases is far worse than the case of always maintaining a relatively higher proportion (≥0.66) of wearing masks.

## Introduction

Emerging infectious diseases (EID) generally happen suddenly, spread rapidly, and have another characteristic of the high incidence of susceptibility crowd. Since the 1970s, EID have appeared almost every year, which has a huge impact on human health and the order of social life. So EID have become a key concern in the global public health field [1]. The infectious disease COVID-19 caused by a novel coronavirus which broke out at the end of 2019 in Wuhan, China, is different from the previous cases. It is contagious in the latent period, and its transmission speed is faster than that of other infectious diseases. Therefore, the research on such an infectious disease has become particularly important. Wu et al. [2] provide the evidence that asymptomatic virus carriers may be potential sources of COVID-19 virus. Shao et al. [3] and Lauer et al. [4] point out the importance of asymptomatic virus carriers during the transmission of COVID-19. Backer et al. [5] estimate the mean incubation period of COVID-19 virus by investigating the travel history and symptom onset of the early 88 confirmed cases of COVID-19 outside Wuhan. Based on the SEIR model, Zhou et al. [6] infer that COVID-19 is a controllable infectious disease with moderate-high transmissibility, and thus more timely and effectively control measures are required to curb further transmission of the epidemic. Boccaletti et al. [7] mention that the modeling and prediction of infectious diseases play an important role in the prevention and control of the epidemic and even the end of pandemic. Naik et al. [8] provide a mathematical analysis of a fractional model in the case of no effective vaccine at present using the real data of COVID-19 in Pakistan. Khan et al. [9] establish a mathematical model of COVID-19 with quarantine and isolation and obtain the stability as well as the corresponding basic reproductive number in the Atangana–Baleanu sense. Fatmawati et al. [10] observe a number of tuberculosis infection decreases with the decrease of the fractional order by establishing fractional compartment model of tuberculosis. Owusu et al. [11] raise a delay differential equation model to delve into structured treatment interruptions within Ghanaian HIV population and carry out relevant mathematical analysis. Zhang et al. [12] provide a rigorous mathematical analysis of an age-structure model of host virus infection with cell-cell transmission and general humoral immune response. Relevant numerical simulations confirm the theoretical results. Danane et al. [13] present a fractional differential equation model to describe the dynamics of hepatitis B virus infection, and they discover that the order of fractional derivative has no effect on the stability of all equilibria from both the theoretical and the numerical results. Due to the unpredictable nature of responsible pathogen, such as the viruses causing SARS, A/H1N1, Ebola, and COVID-19, the relevant countermeasures to mitigate the outbreak are different. During the outbreak of SARS, people take self-protective measures or non-pharmaceutical intervention measures such as wearing masks, hand-washing, and avoiding public transport and crowded places in Hong Kong [14, 15] and Beijing [16]. In 2009, A/H1N1 influenza pandemic triggered a significant proportion of the population to adjust their behavior and take preventive measures such as social distancing and campus quarantine [17]. Atangana et al. [18] establish a model to describe the spread of Ebola hemorrhagic fever, a deadly disease, in West African countries and reveal that good preventive measures are of great significance to the survival of the whole country. Xiao et al. [19] develop a multi-scale model by linking individual movement to pathogen’s diffusion, find the phenomenon of clustering infection during a disease outbreak, and indicate the need for control measures such as improving air circulation or environmental hygiene. Tang et al. develop a dynamic propagation model to estimate the risk of COVID-19 virus transmission [20] and then update this model to suggest that persistent and strict self-isolation is the best measure [21]. In another paper, Tang et al. [22] indicate that improving the detection rate is also important to mitigate the spread of the disease. Kabunga et al. [23] obtain the basic reproductive number and indicate that monitoring of contacts, detection of latent cells, and treatment can effectively control the spread of the disease. By constructing a fractal COVID-19 model, Goufo et al. [24] find the transfer of the epicenter of COVID-19 virus and conclude that winter is favorable for the spread of coronavirus. Chan et al. [25] offer the first conclusive evidence of person-to-person transmission of COVID-19 and mention that a child is protected from infection by wearing a surgical mask most of the time. On March 18, 2020, the National Health Commission, PRC issued a document [26] to interpret “Notice on publication of scientific guidelines on wearing masks for the public”, in which the importance of masks in the prevention and control of the epidemic is emphasized. Atangana et al. [27] point out the importance of masks in the prevention and control of the epidemic and call on people to wear appropriate masks. In an interview with science magazine, Fu Gao emphasized that wearing a mask cannot only thwart the transmission of COVID-19 virus through droplets, but also prevent the virus from spreading from infected people to others [28].

Considering the existence of infectious diseases with infectiousness in the incubation period, in this paper, we introduce a transmission model of such infectious diseases based on the assumptions that wearing masks could reduce the transmission rate of asymptomatic virus carriers but cannot make the probability of infection in the susceptible population lower and all symptomatic infected individuals wear masks. We study the influences of wearing masks in the susceptible and asymptomatic virus carriers on the epidemic.

This paper is organized in the following way. An infectious diseases transmission model of wearing masks in the susceptible and asymptomatic virus carriers is introduced in Sect. 2. Section 3 offers the relevant mathematical analysis. Section 4 gives numerical results and analysis, while conclusions are presented in Sect. 5.

## Model

**Figure 1:**
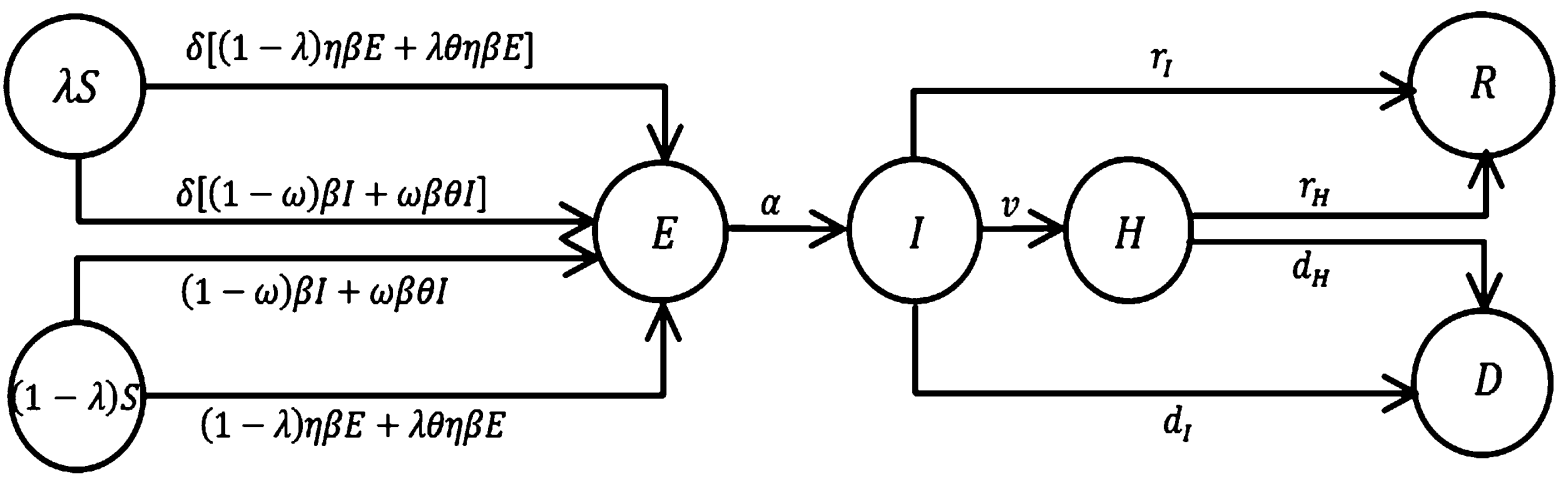
Diagram of transformation relationships of various compartments

In this infectious disease transmission model, we divide the population into six compartments: susceptible $(S)$; carrying the virus but asymptomatic in the incubation period, i.e., exposed $(E)$; infectious with symptoms but not yet isolated, i.e., symptomatic infected $(I)$; hospitalized $(H)$; recovered $(R)$, and death $(D)$. Both *E* and *I* are infection sources because exposed $(E)$ individuals can infect others during the incubation period. We assume that there is no injection of the susceptible population, and the population distribution is even, and people outside the hospital have the same contact rate throughout the whole process. The probability of infection in the susceptible population is independent of age, gender, and other factors, only contact infection is considered here. *S* will be infected through sufficient contact with *E* or *I*, and the probability of infection is *β*. We also assume that the transmissibility of *E* is *η* times that of *I*, where $0\leq \eta \leq 1$ [29, 30]. The mean latent period of this infectious disease is denoted as $\frac{1}{\alpha }$ and the infection period is denoted as $\frac{1}{v}$. For the transformation relationships of the six compartments, see Fig. 1.

Based on the above transformation diagram, the differential equations of the model can be represented as follows:

2.1 $$\begin{aligned} \textstyle\begin{cases} \dot{S}=-\beta S(1-\lambda +\lambda \delta )[(1-\lambda +\lambda \theta )\eta E+(1-\omega +\omega \theta )I], \\ \dot{E}=\beta S(1-\lambda +\lambda \delta )[(1-\lambda + \lambda \theta )\eta E+(1-\omega +\omega \theta )I]-\alpha E, \\ \dot{I}=\alpha E-vI-r_{I}I-d_{I}I, \\ \dot{H}=vI-r_{H}H-d_{H}H, \\ \dot{R}=r_{I}I+r_{H}H, \\ \dot{D}=d_{I}I+d_{H}H. \end{cases}\displaystyle \end{aligned}$$

Other parameters in model (2.1) are summarized in Table 1.

**Table 1:** Descriptions of parameters

Parameters	Descriptions	Range
*β*	Probability of transmission	
*α*	Transition rate of *E* to *I*	[0,1]
*η*	Ratio of the infection rate of exported individuals to the infection rate of symptomatic infected individuals	[0,1]
*λ*	Proportion of masks worn by the susceptible population and the exported population	[0,1]
*ω*	Proportion of masks worn by symptomatic infected individuals	[0,1]
*δ*	Discount on the probability of infection of the susceptible population after wearing masks	[0,1]
*θ*	Discount on the infection rate of the infection sources after wearing masks	[0,1]
*v*	Hospitalization isolation rate of symptomatic infected individuals	[0,1]
$r_{I}$	Recovery rate of symptomatic infected individuals	
$d_{I}$	Death rate of symptomatic infected individuals	
$r_{H}$	Recovery rate of hospitalized patients	
$d_{H}$	Death rate of hospitalized patients	

## Model analysis

The initial data of model (2.1) are given by

3.1 $$\begin{aligned} \begin{aligned} &S(0)=S_{0}>0,\qquad E(0)=E_{0}\geq 0,\qquad I(0)=I_{0}\geq 0,\qquad H(0)=H_{0} \geq 0, \\ &R(0)=R_{0}\geq 0\quad \text{and} \quad D(0)=D_{0}\geq 0. \end{aligned} \end{aligned}$$

For brevity, let $a=\beta (1-\lambda +\lambda \delta )(1-\lambda +\lambda \theta ) \eta $, $b=\beta (1-\lambda +\lambda \delta )(1-\omega +\omega \theta )$. Then the first two equations of (2.1) can be rewritten as

$$\begin{aligned} \textstyle\begin{cases} \dot{S}=-S(aE+bI), \\ \dot{E}=S(aE+bI)-\alpha E. \end{cases}\displaystyle \end{aligned}$$

### Boundedness and positivity of solution

Define $N(t)=S(t)+E(t)+I(t)+H(t)+R(t)+D(t)$ and $\Omega =\{(S, E, I, H, R, D)| S, E, I, H, R, D \ge 0, 0\le N(t)\le N(0)\}$.

#### Lemma 3.1

*Assume that* $(S, E, I, H, R, D)$ *is a solution of* (2.1), *then it is nonnegative and bounded*. *Besides*, *the feasible biologically region given by* Ω *is positively invariant under conditions* (3.1).

#### Proof

From the first equation of (2.1), we know that

$$\begin{aligned} S(t)=S(0)\exp \biggl\{ - \int _{0}^{t}\bigl(aE(s)+bI(s)\bigr)\,ds\biggr\} >0,\quad \forall t>0. \end{aligned}$$

For the case $E(0)>0, I(0)\ge 0$, assume that there exists $t_{1}>0$ such that $E(t_{1})=0$ and $E(t)>0, \forall t< t_{1}$. It follows from the third equation of (2.1) with some simple calculations that

3.2 $$\begin{aligned} I(t)=e^{-(v+r_{I}+d_{I})t} \biggl(I(0)+\alpha \int _{0}^{t}e^{(v+r_{I}+d_{I})s}E(s)\,ds \biggr)>0,\quad \forall 0< t\le t_{1}. \end{aligned}$$

Then we have

3.3 $$\begin{aligned} \bigl(e^{\alpha t}E(t) \bigr)'=e^{\alpha t}S(t) \bigl(aE(t)+bI(t) \bigr)>0, \quad\forall 0< t\le t_{1}, \end{aligned}$$

which yields $E(t_{1})>E(0)e^{-\alpha t_{1}}>0$, a contradiction with $E(t_{1})=0$. Thus, we have $E(t)>0$ for all $t\ge 0$, and $I(t)>0$ for all $t>0$ due to (3.2). From the fourth, the fifth, and the sixth equations of (2.1), it is easy to check that $H(t),R(t),D(t)>0$ for all $t>0$ if $I(t)>0$.

For the case $I(0)>0, E(0)=0$, then $aE(0)+bI(0)>0$. Assume that there exists $t_{1}>0$ such that $aE(t_{1})+bI(t_{1})=0$ and $aE(t)+bI(t)>0, \forall t< t_{1}$. Use (3.3) again to have $E(t)>E(0)e^{-\alpha t}=0$ for all $0< t\le t_{1}$, which also implies $I(t)>0$ for all $0< t\le t_{1}$ by (3.2). This contradicts $aE(t_{1})+bI(t_{1})=0$. So, we obtain that $aE(t)+bI(t)>0, \forall t>0$, and thus $E(t)>0$ by (3.3), $I(t)>0$ by (3.2). It follows from the fourth, the fifth, and the sixth equations of (2.1) that $H(t),R(t),D(t)>0$ for all $t>0$.

For the case $I(0)=E(0)=0$, $(S(0),I(0),E(0))$ is the equilibrium of the first three equations of (2.1). Then $(S(0),0,0,H(0)e^{-(d_{H}+r_{H})t},R(0)+ \frac{r_{H} H(0)}{d_{H}+r_{H}}(1-e^{-(d_{H}+r_{H})t}),D(0)+ \frac{d_{H} H(0)}{d_{H}+r_{H}}(1-e^{-(d_{H}+r_{H})t}) )$ is the corresponding solution.

Adding all the equations of (2.1), we obtain $\frac{dN(t)}{dt}=0$. So, $N(t)\equiv N(0)$, which means that the conservation law is true in the system. And thus $0\le S(t), E(t), I(t), H(t), R(t), D(t)\le N(0)$ due to the positivity of themselves. In addition, the feasible biologically region given by Ω is positively invariant. □

### Existence and uniqueness of solution

Define $X(t)= (S(t), E(t), I(t), H(t), R(t), D(t) )^{T}$, $G(X)= (-S(aE+bI),S(aE+bI),0,0,0,0 )^{T}$, and

$$P= \begin{bmatrix} 0 & 0 & 0 & 0 & 0 & 0 \\ 0 & -\alpha & 0 & 0 & 0 & 0 \\ 0 & \alpha & -(v+d_{I}+r_{I}) & 0 & 0 & 0 \\ 0 & 0 & v & -(r_{H}+d_{H}) & 0 & 0 \\ 0 & 0 & r_{I} & r_{H} & 0 & 0 \\ 0 & 0 & d_{I} & d_{H} & 0 & 0 \end{bmatrix}. $$

Then model (2.1) can be rewritten as follows:

3.4 $$\begin{aligned} X'(t)=PX(t)+G(X). \end{aligned}$$

#### Theorem 1

*There is a unique solution of model* (3.4) *with initial conditions* (3.1).

#### Proof

Let us define the matrix norm by $\||\cdot\||$, where for $P\in \mathbb{R}^{6}\times \mathbb{R}^{6}$: $\||P\||=\rho (P)$, $\rho (P)$ is the spectral norm of *P*. Firstly, the function $X(t)\in R_{6} \rightarrow PX+G(X)$ is continuous. Let $Y(t)=(S'(t), E'(t), I'(t), H'(t), R'(t), D'(t))^{T}$. Then

$$\begin{aligned} &{ \bigl\Vert PX-PY+G(X)-G(Y ) \bigr\Vert }_{R_{6}}\\ &\quad\le \bigl\| \bigl|P\bigr\| \bigr| { \Vert X-Y \Vert }_{R_{6}}+2a \vert E \vert \bigl\vert S-S' \bigr\vert \\ &\qquad{} +2a \bigl\vert S' \bigr\vert \bigl\vert E-E' \bigr\vert +2b \vert I \vert \bigl\vert S-S' \bigr\vert +2b \bigl\vert S' \bigr\vert \bigl\vert I-I' \bigr\vert \\ &\quad\le \max \{\alpha , v+r_{I}+d_{I}, r_{H}+d_{H} \}{ \Vert X-Y \Vert }_{R_{6}} \\ &\qquad{} +2 \max \{a, b\}\max \bigl\{ \vert E \vert , \bigl\vert S' \bigr\vert , \vert I \vert \bigr\} { \Vert X-Y \Vert }_{R_{6}} \\ &\quad\le \max \bigl\{ \alpha , v+r_{I}+d_{I}, r_{H}+d_{H}, \\ & \qquad 2\max \{a, b\}\max \bigl\{ \vert E \vert , \bigl\vert S' \bigr\vert , \vert I \vert \bigr\} \bigr\} { \Vert X-Y \Vert }_{R_{6}}. \end{aligned}$$

And thus, the Cauchy–Lipschitz is satisfied. Consequently, model (3.4) possesses a unique solution [31]. □

### Stability of the disease-free equilibrium ($DFE$) and basic reproduction number $R_{0}$

In this subsection, the disease-free equilibrium ($DFE$) of model (2.1) is given by $P_{0}$. We obtain the basic reproduction number of model (2.1) using the next generation approach [32]. The nonzero equilibrium of model (2.1) is $\{S^{*}, 0, 0, 0, R^{*}, D^{*}\}$. The $DFE$ is given by $P_{0}=\{S^{*}, 0, 0, 0, 0, 0\}$ with $S^{*}=N(0)$. From the positivity of solution and the first equation of model (2.1), we have $\frac{dS}{dt}<0$. So $S(t)< S(0)\le N(0)(t>0)$, which means $P_{0}$ is unstable.

Let $X=(E, I)^{T}$. The matrix of new infections and the transfer matrix between the equations of infections are represented by $F(X)$ and $V(X)$ respectively with

$$\begin{aligned} &F(X)= \begin{bmatrix} \beta S(1-\lambda +\lambda \delta )[(1-\lambda +\lambda \theta )\eta E+(1- \omega +\omega \theta )I] \\ 0 \end{bmatrix} , \\ &V(X)= \begin{bmatrix} \alpha E \\ -\alpha E+vI+r_{I}I+d_{I}I \end{bmatrix} . \end{aligned}$$

The Jacobian matrices of $F(X)$ and $V(X)$ at $P_{0}$ are

$$\begin{aligned} &F(P_{0})= \begin{bmatrix} \beta S^{*}(1-\lambda +\lambda \delta )(1-\lambda +\lambda \theta ) \eta & \beta S^{*}(1-\lambda +\lambda \delta )(1-\omega +\omega \theta ) \\ 0 & 0 \end{bmatrix} ,\\ & V(P_{0})= \begin{bmatrix} \alpha & 0 \\ -\alpha & v+r_{I}+d_{I} \end{bmatrix} . \end{aligned}$$

Thus, the next generation matrix of model (2.1) is

$$FV^{-1}= \begin{bmatrix} \frac{A}{\alpha }+\frac{B}{v+r_{I}+d_{I}} & \frac{B}{v+r_{I}+d_{I}} \\ 0 & 0 \end{bmatrix}, $$

where

3.5 $$\begin{aligned} A=\beta S^{*}(1-\lambda +\lambda \delta ) (1-\lambda + \lambda \theta ) \eta ,\qquad B=\beta S^{*}(1-\lambda +\lambda \delta ) (1- \omega +\omega \theta ). \end{aligned}$$

So, the maximum spectral radius of $FV^{-1}$ is given by

$$\begin{aligned} \rho \bigl(FV^{-1}\bigr)=\frac{A}{\alpha }+\frac{B}{v+r_{I}+d_{I}}=R_{0}, \end{aligned}$$

which is also known as the basic reproduction number.

## Results and discussion

In this section, the numerical simulations are considered. Take $S(0)=10\text{,}000\text{,}000, E(0)=1, I(0)=0, H(0)=0, R(0)=0, D(0)=0$. The relevant parameters quoted and estimated based on the epidemic data of COVID-19 in Wuhan, China [33] are used as an example to study such an infectious disease. According to the method of [34] and the actual epidemic data of Wuhan [33], we choose $\beta =6\times 10^{-8}$. Some studies have illustrated that the median of the incubation period is four days [35, 36], so $\alpha =\frac{1}{4}$. Since the symptoms of such an infectious disease are similar to the common cold and because of insufficient awareness of such an infectious disease, there is a mean 5-day delay from symptom onset to detection/hospitalization of a case [37–39]. In our model, we set the infectious period is five days. Therefore, the confirmed rate is $v=0.2$.

The primary aim of this paper is to study the influences of wearing masks in the susceptible and exported populations on the epidemic. Therefore, we assume that all symptomatic infected individuals wear masks ($\omega =1$), the probability of infection of the susceptible does not decrease after wearing masks ($\delta =1$). The discount $\theta (0<\theta <1)$ on the infection rate caused by wearing masks can be seen as the protective effect of the mask. Take $\theta =0.3, 0.4, 0.5, 0.6, 0.7$. Because the infection rate of *E* is lower than that of *I* [29, 30], we choose $\eta =0.4$. Two situations of the proportion of wearing masks in the susceptible and exported populations are considered here.

### $R_{0}$

Based on the values of the above related parameters, when no one wears a mask ($\lambda =\omega =0$) or the mask has no effect ($\theta =1$), we obtain $R_{0} \approx 3.569$. When all people are wearing effective masks ($\lambda =\omega =1, \theta \neq 1$), $R_{0} \approx 3.569\theta $. It shows that the higher the protective effect of the mask, the more effective it is to control the development of the epidemic. The result also reflects the importance of masks in reducing infectiousness of this infectious disease.

### Constant *λ*

**Figure 2:**
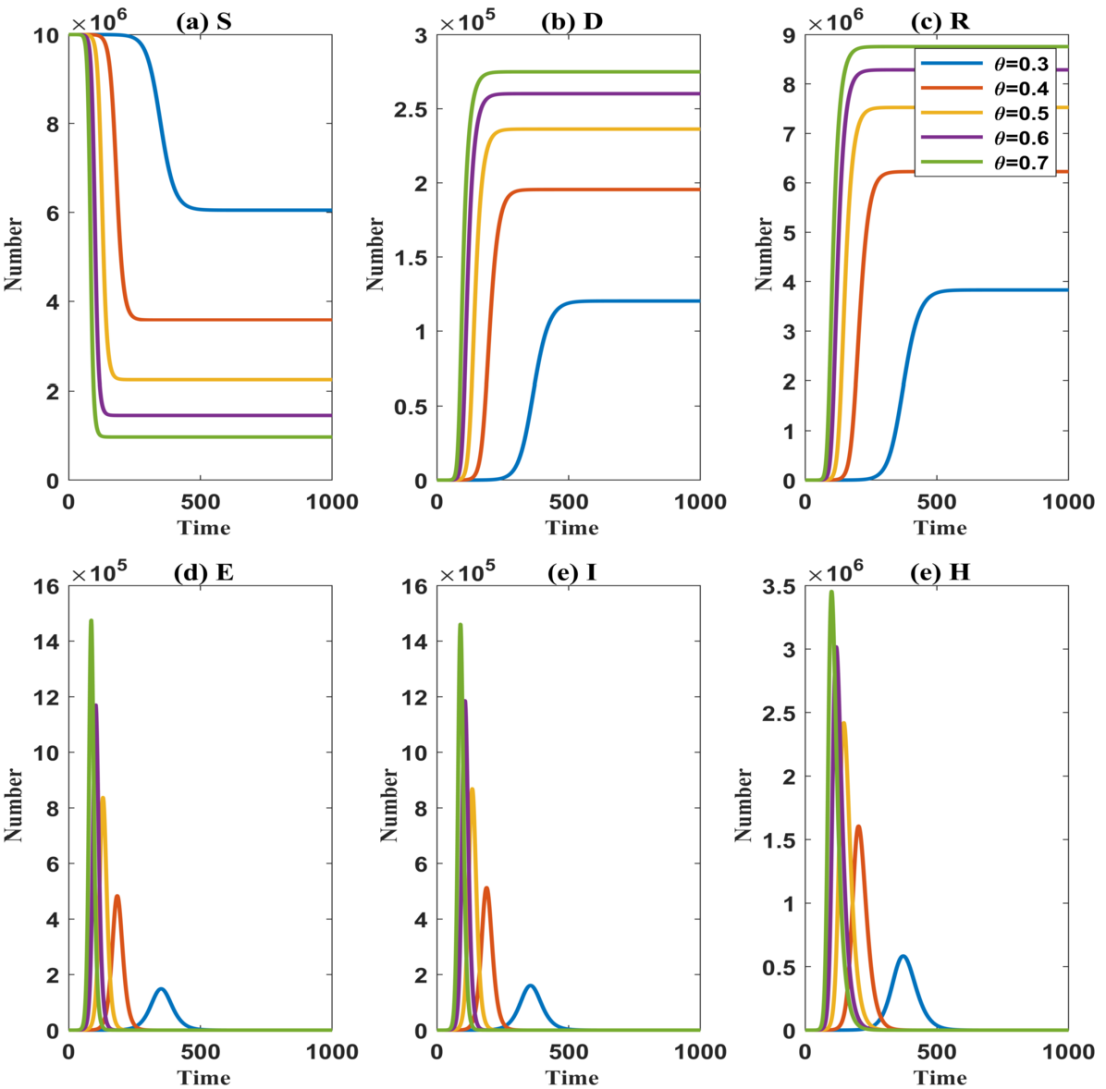
The evolution of the susceptible population $(S)$, the exported population $(E)$, the symptomatic infected population $(I)$, the hospitalized population $(H)$, the recovered population $(R)$, and the death population $(D)$ over time under $\theta =0.3, 0.4, 0.5, 0.6, 0.7$. (**a**): the number of *S*; (**b**): the number of *D*; (**c**): the number of *R*; (**d**): the number of *E*; (**e**): the number of *I*; (**f**): the number of *H*

One situation is that the proportion *λ* of masks worn by susceptible people and exposed people does not change with time, i.e., *λ* is a constant. When the susceptible and exported individuals start to wear masks on the first day and $\lambda =0.7$, the evolution of various compartments over time is shown in Fig. 2.

Based on this group of parameters, when *θ* is different, the numerical examples presented in Fig. 2(a), (b), (c) indicate that the susceptible population, the death population, and the recovered population all tend to be stable. The difference between the initial value of the susceptible population (i.e., $S(0)=10^{7}$) and the final stable value of the susceptible population is an important research object in our next study, defined as the final number of the infected population. It can be seen that the smaller the *θ*, the smaller the stable values of the death population and the recovered population, the greater the stable value of susceptible individuals, and then the smaller the stable value of the infected population, but the later the time that they reach steady states. It is shown that the smaller the discount on the infection rate of infection sources after wearing masks, the larger the reduction in the final number of the infected population and the death toll.

The numerical results in Fig. 2(d), (e), (f) show that there are peaks in the exported population $(E)$, the symptomatic infected population $(I)$, and the hospitalized population $(H)$, respectively. The smaller the *θ* is, the lower the peaks and the later the times when the peaks appear are. Moreover, for each *θ*, the peak of *H* appears later and the value is larger than that of *E* and *I*. In fact, the peak value of *H* is very important for medical capabilities. The ending time of the epidemic is defined as the time when $E=I=0$. As shown in Fig. 2(e), when $\theta =0.3$, 0.4, 0.5, 0.6, 0.7, the epidemic will end on the 728th day, the 402th day, the 296th day, the 242th day, and the 209th day, respectively, which implies that the stronger the protective effect of the mask, the longer the duration of the epidemic. So the phenomenon that the duration of the epidemic is prolonged in low prevalence appears.

**Figure 3:**
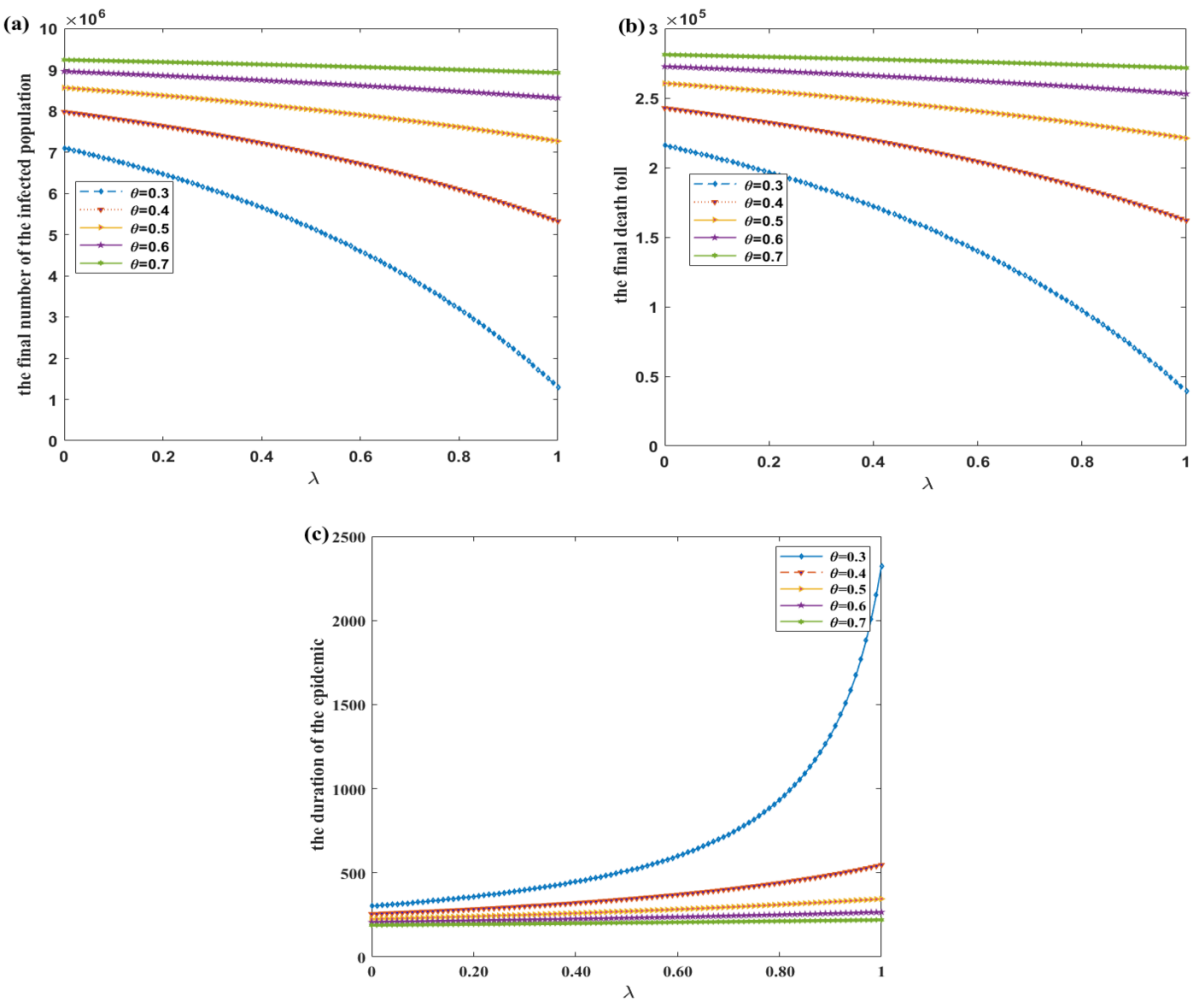
The influences of different proportions of wearing masks in the susceptible and exported populations on the final number of the infected population, the final death toll, and the duration of the epidemic under $\theta =0.3, 0.4, 0.5, 0.6, 0.7$. Vertical axis: (**a**): the final number of the infected population; (**b**): the final death toll; (**c**): the duration of the epidemic. Lateral axis: the proportion *λ* of wearing masks in the susceptible and exported populations

To study the effects of different proportions of wearing masks in the susceptible and exported populations (i.e., *λ*) on the epidemic, we examine the changes of the final number of the infected population, the final death toll, and the duration of the epidemic in Fig. 3 when susceptible and exported individuals start to wear masks on the first day and $\eta =0.4$.

As shown in Fig. 3(a), when $\theta =0.3, 0.4, 0.5, 0.6, 0.7$, the larger the *λ*, the lower the final number of the infected population. Compared with the case $\lambda =0$, when $\lambda =1$, the reduction in the final number of the infected population is 5.807 million, 2.656 million, 1.296 million, 0.645 million, and 0.314 million, a decrease of 81.83%, 33.29%, 15.14%, 7.20%, and 3.39%, respectively. When $\lambda =0.7$, the final number of the infected population is 3.950 million, 6.421 million, 7.761 million, 8.546 million, and 9.031 million, respectively. The final number of the infected population reduces by 44.34%, 19.51%, 9.34%, 4.61%, and 2.24%, respectively.

It is important to note that we assume wearing masks could not reduce the probability of infection in the susceptible population here. Therefore, the reason why the final number of the infected population decreases is that the increase of *λ* makes the transmission rate of this infectious disease virus reduced. Moreover, the stronger the protective effect of the mask, the more reduction in the final number of the infected population.

As shown in Fig. 3(b), when $\theta =0.3, 0.4, 0.5, 0.6, 0.7$, the larger the *λ*, the lower the final death toll. Compared with the case $\lambda =0$, when $\lambda =1$, the reduction in the final death toll is 176,733, 80,827, 39,457, 19,631, and 9543, a decrease of 81.83%, 33.29%, 15.14%, 7.20%, and 3.39%, respectively. When $\lambda =0.7$, the final death toll is 120,214, 195,431, 236,208, 260,101, and 274,858, respectively. The final death toll reduces by 44.34%, 19.51%, 9.34%, 4.61%, and 2.24%, respectively.

As seen in Fig. 3(a), (b), we find an important information that the quality of masks, which is negative correlation with *θ*, is very important for the effect on preventing and controlling the epidemic. This coincides with the result $R_{0}\approx 3.569\theta $. According to the numerical simulation results, when the quality of the mask is improved such that *θ* changes from 0.4 to 0.3, compared with the situation without mask ($\lambda =0$), if both *S* and *E* wear masks ($\lambda =1$), the reduction ratio of the final number of the infected population and the death toll increases from 33.29% to 81.83%, a great improvement in the effect on preventing and controlling the epidemic. Therefore, when people are required to wear masks during the epidemic, the government also needs to control the quality of masks in circulation strictly.

In Fig. 3(c), when $\theta =0.3, 0.4, 0.5, 0.6, 0.7$, the larger the *λ*, the longer the duration of the epidemic. When *λ* changes from 0 to 1, the increase in the duration of the epidemic is 2020 days, 292 days, 120 days, 60 days, and 31 days, a rise of 6.6887 times, 1.1451 times, 0.5333 times, 0.2913 times, and 0.1640 times, respectively. The negative relation between duration and peaks of this model implies that wearing a mask can reduce the peak value of the symptomatic infected population, and the stronger the protective effect of the mask, the lower the peak and the later the peak appears. That is, the epidemic will slowly end at low prevalence.

Under the assumption that all the symptomatic infected individuals wear masks and the probability of infection of the susceptible population is not reduced after wearing masks as long as the susceptible and exported individuals wear masks, the final number of the infected population and the death toll would be reduced as shown in Fig. 3. This is because there is no difference in the proportion of wearing masks in the exported population and the susceptible population, and wearing masks can reduce the infection rate of the exported individuals, then the final number of the infected population and the death toll are correspondingly decreased. However, as the proportion of masks worn by the susceptible and exported populations increases, the duration of the epidemic increases. This is an extension of the duration of the epidemic with a low prevalence.

**Figure 4:**
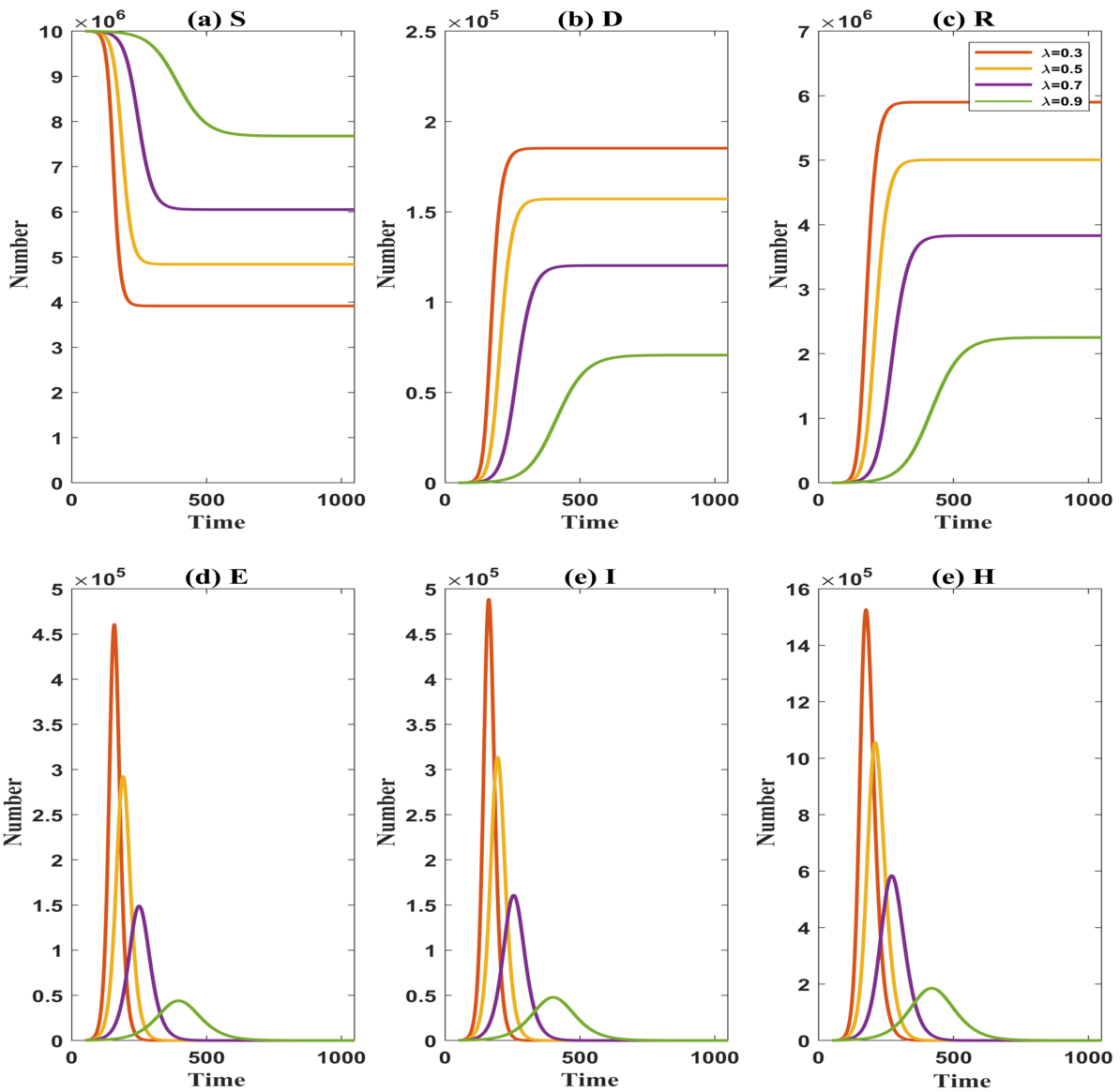
The evolution of the susceptible population $(S)$, the exported population $(E)$, the symptomatic infected population $(I)$, the hospitalized population $(H)$, the recovered population $(R)$, and the death population $(D)$ over time with $\lambda =0.3, 0.5, 0.7, 0.9$. (**a**): the number of *S*; (**b**): the number of *D*; (**c**): the number of *R*; (**d**): the number of *E*; (**e**): the number of *I*; (**f**): the number of *H*

In the early stage, it is not natural for the susceptible and exported populations to wear masks because of insufficient awareness of the epidemic or cultural differences. With the awareness of such an infectious disease increases, one knows that wearing masks can curb the spread of such an infectious disease, thus there are more regulations and voices in wearing masks for public. Assume $\theta =0.3$. Let $t_{1}$ be the time when the susceptible and exported individuals’ awareness of wearing masks begins to awaken. Before $t_{1}$, all susceptible and exported populations do not wear masks $(\lambda =0)$, but after $t_{1}$, the proportion of wearing masks in the susceptible and exported populations can achieve $0.3, 0.5, 0.7, 0.9$. When $t_{1}=50$, the changes of various compartments are presented in Fig. 4.

It can be seen from Fig. 4(a), (b), (c) that with the increase of *λ*, when the system reaches stability, the death population and the recovered population are smaller and smaller, the susceptible becomes larger and larger, so that the final number of the infected population becomes smaller and smaller. However, the times that their stable values appear are negatively correlated with *λ*. Compared the case $\lambda =0.7$ in Fig. 4(a), (b), (c) with the case $\theta =0.3$ in Fig. 2(a), (b), (c), since the time to wear masks is delayed, the stable values of the recovered population and the death population have a slight increase, while the stable value of the susceptible population declines a little. Correspondingly, the stable value of the infected population increases slightly.

As shown in Fig. 4(d), (e), (f), with the increase of *λ*, the peaks of the exported population $(E)$, the symptomatic infected population $(I)$, and the hospitalized population $(H)$ become lower and lower. The times that their peaks appear become later and later. Besides, the duration of the epidemic is longer and longer. The reason is that a high proportion of wearing masks in the susceptible and exported populations makes the infectivity of virus carriers reduced. Compared the case $\lambda =0.7$ in Fig. 4(e) with the case $\theta =0.3$ and $\lambda =0.7$ in Fig. 2(e), the peak of symptomatic infected individuals appears earlier, and the length of the epidemic decreases since the time to start wearing masks is delayed.

**Figure 5:**
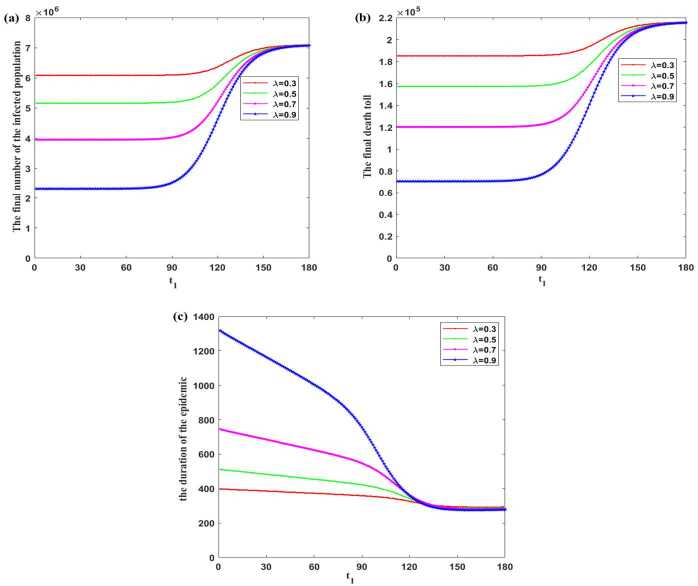
Effects of different moments that the susceptible and exported populations start to wear masks on the final number of the infected population, the final death toll, and the duration of the epidemic under $\delta =1$, $\eta =0.4$, $\theta =0.3, \lambda =0.3, 0.5, 0.7, 0.9$. Vertical axis: (**a**): the final number of the infected population; (**b**): the final death toll; (**c**): the duration of the epidemic. Lateral axis: The moment that the susceptible and exported populations start to wear masks ($t_{1}$)

Next, we discuss the impacts of $t_{1}$ on the final number of the infected population, the final death toll, and the duration of the epidemic. Take $\lambda =0.3, 0.5, 0.7$, and 0.9, respectively. The final number of the infected population, the final death toll, and the duration of the epidemic are shown in Fig. 5.

In Fig. 5(a), the larger the *λ*, the smaller the corresponding final number of the infected population for different $t_{1}$. It is notable that with the increase of $t_{1}$, the final number of the infected population presents slow increase first, then rapid increase, and then slow increase. In the second stage, $t_{1}$ has a very obvious impact on the final number of the infected population. Even if $t_{1}$ is delayed by one day, the final number of the infected population would increase a lot. The larger the *λ*, the earlier the beginning time of the second stage and the greater the rate of increase in the final number of the infected population. In the third stage, no matter what the value of *λ* is, the final number of the infected population will not change significantly. It means that if it is too late to wear a mask, the best period to contain the epidemic has already been missed. Therefore, the sooner we protect ourselves, the more effective is reducing the number of the infected population.

The later the susceptible and exported persons start to wear masks, the higher the final death toll as shown in Fig. 5(b). The larger the *λ*, the lower the corresponding final death toll for each $t_{1}$. A conclusion similar to that of the final number of the infected population is obtained. That is, in the second stage, $t_{1}$ has a very obvious impact on the final death toll. Even if $t_{1}$ is delayed by one day, the final death toll may increase significantly. The larger the *λ*, and with the earlier beginning time of the second stage, the greater the rate of increase in the final death toll. But if the starting time is in the third stage, no matter what *λ* is, the final death toll will not change significantly.

As shown in Fig. 5(c), the later the susceptible and exported persons start to wear masks, the shorter the duration of the epidemic. However, it is worth noting that for each *λ*, there are two corresponding thresholds $t^{*},t^{**}$ such that when $t_{1}< t^{*}$, with the increase of $t_{1}$, the duration of the epidemic slowly reduces, and the larger the *λ*, the greater the reduction in the duration of the epidemic. When $t^{*}< t_{1}< t^{**}$, the duration of the epidemic will be shortened rapidly as $t_{1}$ increases, and the larger the *λ*, the earlier $t^{*}$ appears and the greater the reduction rate of the duration of the epidemic. However, when $t^{**}< t_{1}$, there is almost no difference in the duration of the epidemic with the increase of *λ*. It implies that when $t_{1}$ is too large, the final number of the infected population and the final death toll are very close to the case of $\lambda =0$. Maybe this is because that the natural evolution process of the epidemic has been basically completed, and people have achieved herd immunity at $t^{**}$. Therefore, maintaining a high proportion of wearing masks in the susceptible and exported populations from the beginning of the epidemic could effectively hinder the spread of the disease.

### $\lambda =\lambda (t)$

The other situation is that the proportion of wearing masks in the susceptible and exported populations changes with time (i.e., $\lambda =\lambda (t)$), which can be considered as a reflection of self-awareness. Tang et al. [40] indicated that the classical compartment model without behavior change and the model with average rate of behavior change depicted by an exponential function could fit the observed data better. So we consider the case that the awareness of wearing masks for people changes with the daily briefing of the epidemic.

Assume that $\lambda (t)$ increases with the increase in confirmed new cases and new deaths, which has the following form:

$$\begin{aligned} \lambda (t)=1-k_{1}e^{-k_{2} vI}-(1-k_{1} )e^{-k_{3} d_{H} H}, \end{aligned}$$

where $k_{1}, k_{2}, k_{3}\in [0,1]$. According to the global COVID-19 epidemic statistics released by the World Health Organization from April 18 to May 28, the mean ratio of the confirmed new cases and new deaths is about 20 [41]. Thus, we take $k_{2}=0.005$, $k_{3}=0.1$.

**Figure 6:**
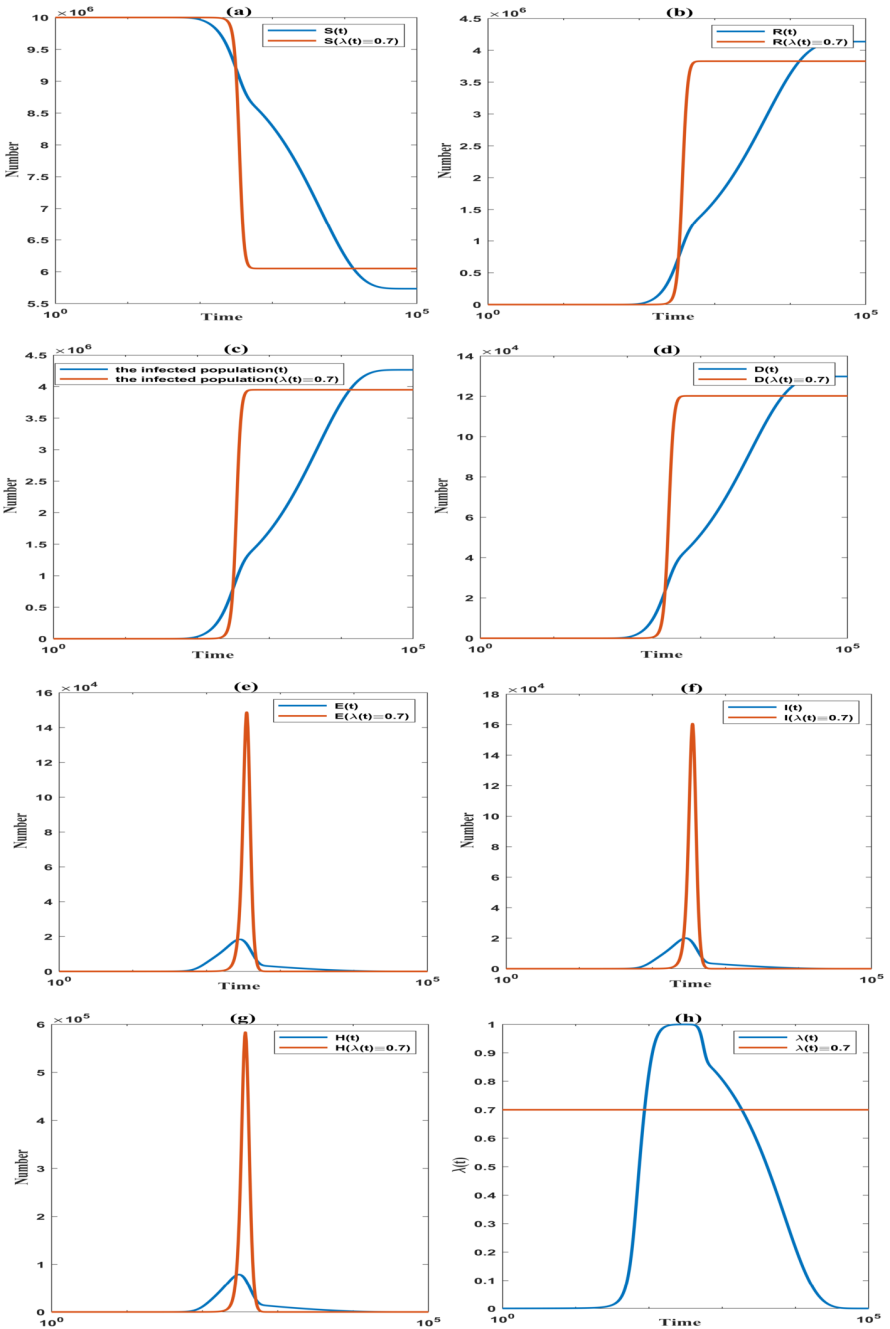
The evolution of the susceptible population $(S)$, the recovered population $(R)$, the infected population, the death population $(D)$, the exported population $(E)$, the symptomatic infected population $(I)$, the hospitalized population $(H)$ for $\lambda (t)\equiv 0.7$ and $\lambda (t)$ changes with time. The proportion of wearing masks in the susceptible and exported populations $\lambda (t)$ over time. Lateral axis: logarithm of time *t*

For the cases $\lambda (t)\equiv 0.7$ and $\lambda (t)$ changes with time, see Fig. 6 for the evolution of various compartments under $k_{1}=0.5$, $t_{1}=1$, and $\theta =0.3$.

The numerical examples presented in Fig. 6(a), (b), (c), (d) indicate that the susceptible population, the recovered population, the infected population, and the death population all tend to be stable as time goes. The stable values of the infected population and the death toll are 4.27 million people and 129,851 people, respectively. Compared with $\lambda (t)\equiv 0.7$, when $\lambda (t)$ changes with time, both the final number of the infected population and the death toll increase 8.02%. Compared with the case $\theta =0.3$ and $\lambda \geq 0.66$ in Fig. 3(a), (b), the final number of the infected population and the death toll would increase significantly, and their maximum growth factors are 2.31.

Compared with $\lambda (t)\equiv 0.7$, when $\lambda (t)$ changes with time, the numerical results in Fig. 6(e), (f), (g) show that the peaks of the exported population $(E)$, the symptomatic infected population $(I)$, and the hospitalized population $(H)$ are evidently reduced. So the pressure of the health care has been greatly reduced. The peak of *I* is lower and comes earlier, but the duration of the epidemic increases 67.07 times, which reflects the fact that the duration of the epidemic lasts longer in the low prevalence. Moreover, in comparison with the case $\theta =0.3$ in Fig. 3(c), the duration of the epidemic has increased 21.03 times at least. This is because $\lambda (t)$ is gradually reduced with the decrease in the confirmed new cases and new deaths, and the long-term coexistence of infectious individuals and the unsustainable susceptible population happened.

In Fig. 6(h), the mean value of $\lambda (t)$ is 0.13, which is very small during the whole epidemic. But during the 100 days before the peak and the 100 days after the peak of the symptomatic infected population, the average of $\lambda (t)$ is 0.9994 which means that almost all the susceptible and exported populations wear masks during these days. Moreover, there are 1772 days when $\lambda (t)$ is greater than 0.7. Nevertheless, compared with $\lambda (t)\equiv 0.7$, the final number of the infected population and the final death toll increase significantly, and the duration of the epidemic also increases 67.07 times. It means that keeping a high and stable proportion of wearing masks in the susceptible and exported populations from the early stage of the epidemic can effectively reduce the final number of the infected population and the final death toll. And thus the epidemic would end as soon as possible.

## Conclusion

In this paper, we study the effects of personal protective measure of wearing masks on the spread of infectious disease with the property that infectiousness happened in the latent period. In previous studies, the exported people who are virus carriers are asymptomatic and non-contagious. However, exported people of COVID-19 are contagious in the incubation period, which motivates us to study the effect of wearing masks in the susceptible and exported populations on the epidemic of this infectious disease. At first, we establish the existence, uniqueness, boundedness, and nonnegativity of the solution as well as the positive invariance of the feasible region, obtain the corresponding disease-free equilibrium point and the expression of the basic reproduction number of model (2.1). Then the numerical simulation results are shown which imply that when all the symptomatic infected individuals wear masks, and wearing masks could not reduce the probability of infection of the susceptible population if the proportion of wearing masks in the susceptible and exported populations is constant, as long as the masks are effective (for example $\theta \le 0.3$), the final number of the infected population and the death toll could be effectively reduced. In addition, the earlier the moment of wearing masks, the better the control of the epidemic. Furthermore, there are two corresponding thresholds. When the starting time of wearing masks is between the two thresholds, even if it is delayed by one day, both the final number of the infected population and the death toll will increase significantly. The higher the proportion of wearing masks in the susceptible and exported populations, the higher the reduction rate of the final number of the infected population and the death toll. However, if the starting time is too late, the final number of the infected population and the death toll almost do not change with the proportion of wearing masks in susceptible and exported populations. Besides, if masks are worn in time, the duration of the epidemic increases as the proportion of wearing masks in the susceptible and exported populations increases. And thus the phenomenon that the duration of the epidemic is prolonged in a low prevalence case is proved. If the proportion of wearing masks in the susceptible and exported populations changes with the confirmed new cases and new deaths, and masks have strong protective effect ($\theta =0.3$) as well, compared with the constant proportion case ($\lambda \in [0.66,1]$), both the final number of the infected population and the death toll increase obviously, and the duration of the epidemic increases in a huge proportion. But there is an interesting phenomenon that the pressure of the health care could be reduced greatly due to the reduction in the hospitalized population *H*. In conclusion, our results indicate that the larger-scale spread of the epidemic can be curbed and sufficient time can be set aside for health professionals and related institutions to formulate relevant diagnosis and treatment plans and develop targeted vaccines by maintaining a relatively high and stable ratio of wearing masks from the early stage of the epidemic.

In this paper, we did not consider the impact of habit, environment, and public opinion on the proportion of wearing mask. In the future, mechanisms that can affect behavior changes will be considered to study the impact of a specific protective measure on the spread of the epidemic.

## Data Availability

Not applicable.
